# Patients with Leptomeningeal Carcinomatosis and Hydrocephalus-Feasibility of Combined Ventriculoperitoneal Shunt and Reservoir Insertion for Intrathecal Chemotherapy

**DOI:** 10.3390/curroncol31050180

**Published:** 2024-04-24

**Authors:** Matthias Schneider, Christian Wispel, Anna-Laura Potthoff, Muriel Heimann, Valeri Borger, Christina Schaub, Ulrich Herrlinger, Hartmut Vatter, Patrick Schuss, Niklas Schäfer

**Affiliations:** 1 Department of Neurosurgery, University Hospital Bonn, 53127 Bonn, Germany; christian.wispel@ukbonn.de (C.W.); anna-laura.potthoff@ukbonn.de (A.-L.P.); muriel.heimann@ukbonn.de (M.H.); valeri.borger@ukbonn.de (V.B.); hartmut.vatter@ukbonn.de (H.V.);; 2 Division of Clinical Neuro-Oncology, Department of Neurology, University Hospital Bonn, 53127 Bonn, Germany; christina.schaub@ukbonn.de (C.S.); ulrich.herrlinger@ukbonn.de (U.H.); niklas.schaefer@ukbonn.de (N.S.)

**Keywords:** brain metastasis, leptomeningeal carcinomatosis, intrathecal chemotherapy, ventriculoperitoneal shunt, hydrocephalus, Rickham reservoir

## Abstract

Therapeutic management of patients with leptomeningeal carcinomatosis (LC) may require treatment of concomitant hydrocephalus (HC) in addition to intrathecal chemotherapy (ITC). Ventriculoperitoneal shunts (VPS) equipped with a valve for manual deactivation of shunt function and a concomitant reservoir for application of ITC pose an elegant solution to both problems. The present study evaluates indication, feasibility, and safety of such a modified shunt/reservoir design (mS/R). All patients with LC aged ≥ 18 years who had undergone mS/R implantation between 2013 and 2020 at the authors’ institution were further analyzed. ITC was indicated following the recommendation of the neuro-oncological tumor board and performed according to a standardized protocol. Sixteen patients with LC underwent mS/R implantation for subsequent ITC and concomitant treatment of HC. Regarding HC-related clinical symptoms, 69% of patients preoperatively exhibited lethargy, 38% cognitive impairment, and 38% (additional) visual disturbances. Postoperatively, 86% of patients achieved subjective improvement of HC-related symptoms. Overall, postoperative complications occurred in three patients (19%). No patient encountered cancer treatment-related complications. The present study describes a combination procedure consisting of a standard VPS-system and a standard reservoir for patients suffering from LC and HC. No cancer treatment-related complications occurred, indicating straightforward handling and thus safety.

## 1. Introduction

During the course of their disease, a substantial proportion of cancer patients develop brain metastases (BM) [1]. With the availability of improved diagnostic tools allowing earlier detection and the emergence of more efficient therapeutic modalities allowing longer systemic control and survival, it seems likely that the incidence of BM will continue to increase [2,3]. Some patients will suffer neurological deficits leading to diagnosis in this setting. In addition, the development of hydrocephalus (HC) with resulting symptoms may also provide rationale for the diagnosis of BM. Nevertheless, leptomeningeal seeding of tumor cells with the formation of linear or nodular leptomeningeal carcinomatosis (LC) may occur in the setting of the underlying malignancy [4]. Such linear LC might also secondarily cause HC by inhibiting adequate cerebrospinal fluid (CSF) absorption due to the proliferation of metastatic cells in the subarachnoid space [4,5,6]. Symptoms of HC, such as headaches, nausea, vomiting, gait disorders, urinary incontinence, cerebral nerve palsy, and even mental changes might interfere with quality of life as well as systemic cancer treatment by worsening the physical situation of the affected patient [7]. The common neurosurgical treatment of HC consists of surgical release of the obstruction in the area of the CSF drainage pathways (via BM resection) or, if this is not possible, then by establishing a permanent subcutaneous CSF drainage into the abdominal space using a ventriculo-peritoneal shunt (VPS) [8].

In addition, individual cases require intrathecal chemotherapy (ITC) as part of the treatment of linear leptomeningeal CNS colonization [4,9]. For this purpose, a separate reservoir for intraventricular injection of therapeutic agents is usually used in patients with linear or nodular LC and positive CSF cytology [4,9,10].

In a few cases, both the treatment of HC and enabling ITC may be necessary. Ventriculoperitoneal shunts (VPS), which are equipped with a valve to manually switch off the shunt function and at the same time with a reservoir for the application of ITC, pose an elegant solution to both problems. Furthermore, modified shunt systems (combined with reservoirs) might also reduce the number of interventions in these critically ill patients [11]. However, surgical intervention in such a highly vulnerable patient cohort may also be associated with a significantly elevated risk profile for peri- and postoperative complications [12], which could negate the originally intended benefit of rapid symptom relief through HC treatment. Rapidly proceeding with surgery in multiple body cavities, such as the brain and abdomen—as is the case with shunt operations—shortly after the conclusion of chemo- and/or radiotherapy might, for instance, carry a high risk for shunt infections due to compromised wound healing from cytotoxic therapies [13]. The urgent shunt implantation in cancer patients with multiple metastases, additional LC, and existing HC is a rare but often unavoidable approach in clinical settings to potentially alleviate symptoms in this challenging situation. Scientific literature on this subject is scarce. Against this backdrop, the present study evaluates the indications, feasibility, and safety of shunt/reservoir constructions in patients with LC and HC.

## 2. Materials and Methods

### 2.1. Patients

The present study was performed in accordance with the Declaration of Helsinki and approved by the institutional ethics committee (No. 250/19). Between 2013 and 2020, 16 patients with the diagnosis of LC who underwent VPS implantation with an additional reservoir were enrolled in this retrospective study. Patients with a primary brain tumor were excluded. All patient records were examined for symptoms, primary site of malignancy, subtype of hydrocephalus, improvement of symptoms after VPS, and postoperative shunt-related complications. BM was diagnosed based on magnetic resonance imaging (MRI) findings, pathological findings, or both. Patients were evaluated at admission based on their clinical-functional constitution using the Karnofsky Performance Score (KPS), categorizing them into either ≥70% or <70%, as previously described [14].

Indication for VPS implantation was discussed in our neurooncological tumor board. In this interdisciplinary conference, neurooncological cases are debated weekly depending on the underlying tumor entity, as previously reported [12,15]. In the case of a necessary intrathecal agent application, implantation of a Rickham reservoir was usually chosen as a convenient, quick-to-perform option. In the case of patients with concomitant signs of hydrocephalus (both clinically and radiologically) and/or suspected swelling during further radiation treatment with consecutive obstruction of relevant CSF drainage, the recommendation for shunt placement was also expressed. These neurosurgical procedures were then performed in a combined fashion.

Upon further investigation, the type of suspected hydrocephalus (HC) was divided into disturbed CSF resorption (communicating HC) or obstruction of CSF flow by tumor mass/further treatment (obstructive HC). Imaging criteria of HC were determined by cranial CT and/or MRI. Clinical symptoms requiring VPS implantation were headache, nausea/vomiting, loss of consciousness, visual disturbances, urinary incontinence, cognitive impairment, gait disorders, and/or cranial nerve paresis.

### 2.2. Operative Procedure

**Figure 1:**
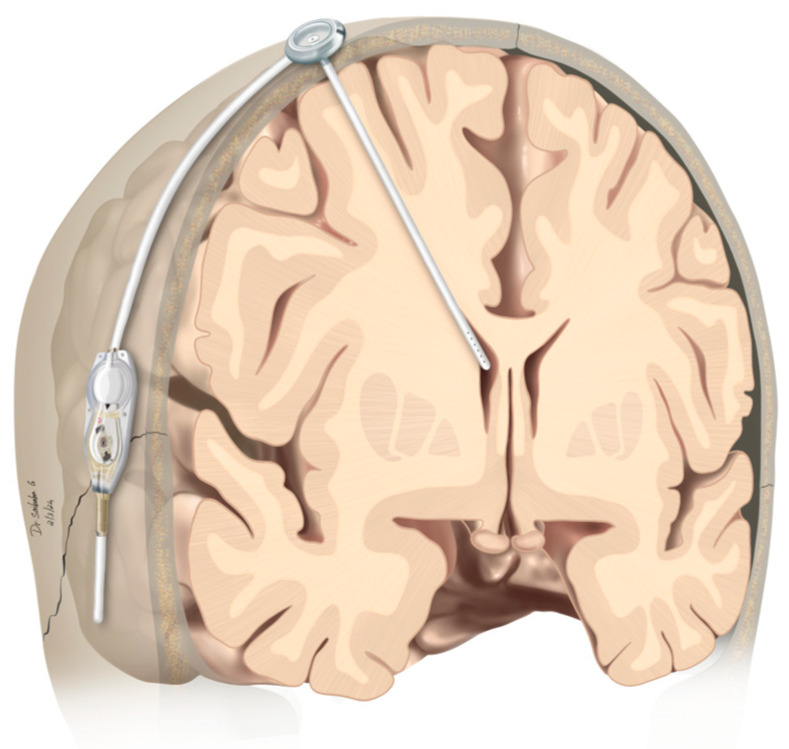
Graphical representation of modified shunt/reservoir construction. The CSF drainage system from the lateral ventricle to the peritoneum includes a programmable valve that allows for the adjustment of the flow rate. Additionally, a Rickham reservoir is integrated into the system, providing easy intrathecal access, such as for the administration of ITC. CSF, cerebrospinal fluid; ITC, intrathecal chemotherapy.

The modified VP-Shunt/reservoir (mS/R)-implantation was performed in a supine position under general anesthesia. The standard approach was on the right side, but was adapted accordingly in case of any previous abdominal operations/scarring. In the first step, the ventricular shunt catheter was connected to a Rickham reservoir, which was then inserted through a burr hole into the frontal horn. The shunt valve was placed retroauricularly (see Figure 1). In the authors’ hospital, Codman Hakim Medos programmable valves (Codman Specialty Surgery, Integra LifeSciences, Plainsboro, NJ, USA) were used until 2015; thereafter, CertasPlus programmable valves (Codman Specialty Surgery, Integra LifeSciences, Plainsboro, NJ, USA) were used for VP shunt implantation. The standard setting for CertasPlus valves was selected as pressure level 5, which reflects a resistance of 145 ± 35 mmH_2_O. The distal shunt catheter was subcutaneously tunneled and inserted periumbilically into the peritoneal cavity [16,17].

Patients were observed postoperatively on a general neurosurgical ward. Correct placement of the ventricular catheter was assessed via cranial CT scan. Correct placement of the distal parts of the VPS and valve adjustment were objectified by X-ray. Afterward, the patients were further cared for by colleagues of the division of neuro-oncology in order to be able to start the ITC promptly after intracranial position control. All postoperative adverse events were categorized according to the therapy-oriented Clavien–Dindo classification system (CDC) [18].

### 2.3. Intrathecal Chemotherapy Protocol

Diagnosis of LC was confirmed in all patients by histopathological evidence using CSF cytology. ITC was indicated following the recommendation of the neurooncological tumor board and performed according to a standardized protocol [19]. The histology of the primary tumor and the type of LC (non-adherent type with or without adherent type with linear or nodular MRI findings) were taken into account when choosing the treatment regimen. Methotrexate was the chemotherapeutic compound used for ITC. ITC was started with injections twice a week for a maximum of 4 weeks and thereafter reduced in dependance of tolerability and response to a weekly or once a month injection. After injection of intrathecal MTX patients received 15 mg oral calcium folinate every 6 h for 48 h.

ITC was injected into the CSF via the Rickham reservoir. The shunt valve was then switched off to avoid immediate drainage of the chemotherapeutic agent into the peritoneal cavity via the shunt system. After 2–6 h the shunt valve was reopened. Patients were monitored clinically in the neuro-oncological ward.

### 2.4. Data Collection and Statistical Analysis

Data collection was gathered using SPSS software for Windows (Version 27, IBM Corp., Armonk, NY, USA). Analysis of categorical data was conducted through Fisher’s exact test for pairs of variables and the chi-square test for scenarios with more than two variables. The Mann–Whitney U-test was utilized for non-normally distributed data comparison. Kaplan–Meier method via GraphPad Prism for MacOS (Version 9.4.1, Graphpad Software, Inc., San Diego, CA, USA) was applied for analyzing overall survival (OS) rates. Significance was assigned to results with *p*-values less than 0.05.

## 3. Results

### 3.1. Patient Characteristics

Between 2013 and 2020, 16 patients with LC underwent mS/R-implantation for subsequent ITC and concomitant treatment of HC. Among these, 4 out of 16 patients (25%) had LC without additional solid intracranial metastases, 3 out of 16 patients (19%) presented with LC and a single solid intracranial metastasis, while 9 out of 16 patients (56%) were diagnosed with LC and multiple solid intracranial metastases. Median age was 58 years (interquartile range [IQR] 50–66) at the time of mS/R surgery. Preoperatively, patients exhibited a median KPS of 50 (IQR 40–60).

The patients with mS/R implantation suffered from the following underlying malignancies: breast cancer in eight patients (50%), gastrointestinal cancer in three patients (19%), lung cancer in three patients (19%), malignant melanoma in one patient (6%), and urogenital cancer in one patient (6%). Median overall survival (OS) for the entire study cohort measured from the day of shunt implantation was 4 months (95% confidence interval (CI) 0–10 months). Further details are given in Table 1.

### 3.2. Hydrocephalus

**Figure 2:**
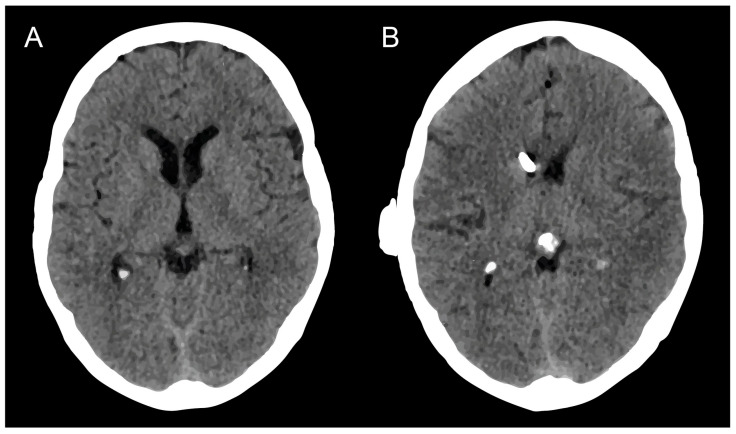
(**A**) Cranial CT scan on the day before surgery and (**B**) cranial CT scan on the postoperative day, showing a reduction in ventricular size in a patient with occlusive HC due to LC. The end of the cranial shunt catheter is visible projecting onto the foramen of Monroi in image (**B**). HC, hydrocephalus; LC, leptomeningeal carcinomatosis.

Regarding concomitant hydrocephalus, nine patients with LC suffered from communicating hydrocephalus (56%). Seven out of sixteen patients (44%) presented with obstructive hydrocephalus. Among these, four patients (57%) had obstructive hydrocephalus due to multiple intracranial metastases. In two of the seven patients (29%), the CT-confirmed obstructive hydrocephalus was caused exclusively by meningeal carcinomatosis, which obstructed the aqueduct. In one patient (14%), a single intracranial metastasis and the accompanying perifocal edema led to hydrocephalus. In all affected patients, a radiological improvement of hydrocephalus could be observed early postoperatively. For an illustrative case, see Figure 2.

Concerning the clinical symptoms, which were preoperatively perceived as being caused by the concomitant HC in 14 patients, 11 patients exhibited preoperative lethargy (69%), 6 suffered from cognitive impairment (38%), whereas 6 patients reported (additional) visual disturbances (38%). Postoperatively, 12 of 14 patients (86%) achieved a subjective improvement in the preoperative complained HC-related symptoms before starting ITC.

### 3.3. Surgical Management of mS/R

The median surgical time for mS/R was 66 min (IQR 37–72) in patients with LC. Overall, 3 of the 16 patients (19%) experienced postoperative complications after mS/R procedures. Postoperative infections were observed in 2 of 16 patients with mS/R surgery (13%). Those patients had been treated with radiotherapy priorly. Wound healing disturbances without infection occurred in 1 of 16 patients (6%). That patient had been treated with ITC and high-dose glucocorticoids priorly. Postoperative mechanical mS/R problems (e.g., catheter dislocation) arose in 2 of 16 patients (13%). In addition, postoperative hygroma requiring treatment occurred in none of the patients with mS/R surgery. Details on postoperative complications, as well as complication severity according to the CDC, are given in Table 2.

### 3.4. Feasibility/Procedure of ITC with mS/R

A total of six patients with mS/R received ITC as planned (38%). In the remaining 10 patients, planned ITC was not administered for the reasons outlined in the following. In six patients, the intraoperatively obtained CSF sample showed no positive cytology, so that ITC was not indicated. The additional implantation of the Rickham reservoir remained without consequences in all of these six patients during further treatment. In two patients, ITC was planned but was not performed because of the rapid deterioration of the patients’ clinical conditions, resulting in a change of the therapeutic strategy to a palliative regimen. Another patient withdrew from the planned ITC during the short-term postoperative course after several informed consent consultations and opted for further palliative treatment. In one patient, ITC was planned but was not performed due to postoperative shunt infection. No patient experienced chemotherapy-associated complications or problems with the adjustment of the on/off shunt valve. All patients received intrathecal administration of the chemotherapeutic agent methotrexate in case of planned ITC (Table 1). After an initial briefing, the treating colleagues from the institutional division of neuro-oncology performed the necessary shunt valve adjustments independently and without any problems. The short-term closure of the VPS system necessary for the application of ITC was well tolerated by all analyzed patients with mS/R without clinical signs of increased intracranial pressure.

## 4. Discussion

The current study offers initial evidence that combining a ventriculoperitoneal shunt system with an adjustable valve and a Rickham reservoir is both feasible and safe for treating terminally ill patients with brain metastases, hydrocephalus, and a need for intrathecal chemotherapy. This strategy effectively minimizes surgical intervention by addressing both hydrocephalus management and chemotherapy delivery in a single procedure. Early postoperative results indicate radiological improvement in hydrocephalus and subjective symptom relief in the majority of patients, with a manageable incidence of complications. The median operation time for the procedure is reasonable, and postoperative care, including shunt valve adjustments, can be independently managed by treating teams. This approach presents a practical option for simultaneously treating hydrocephalus and facilitating intrathecal chemotherapy in this patient population.

Leptomeningeal metastases from solid tumors represent a dire prognostic indicator, typically heralding a terminal phase of cancer with significantly reduced survival rates [20]. The treatment of LM in terminally ill patients is highly individualized, necessitating a thorough consideration of each patient’s extensive medical history and previously attempted therapeutic strategies. This personalized treatment approach is crucial due to the complex interplay between the primary tumor’s characteristics, the extent of metastatic spread, and the patient’s overall health and treatment tolerance. Recent research has further nuanced our understanding of LM by identifying prognostic differences across various LM subtypes. Le Rhun et al. have underscored the importance of cytological analysis in guiding therapeutic decisions, revealing that certain LM subtypes, particularly those with positive cytology, might respond differently to treatment modalities [4]. This differentiation has significant implications for tailoring treatment plans, emphasizing the need for a meticulous assessment of LM characteristics to optimize therapeutic outcomes. In light of these insights, the management of LM in the context of advanced cancer remains a formidable challenge, requiring a judicious balance of aggressive treatment to control disease progression while minimizing adverse effects on patient quality of life.

Patients with leptomeningeal seeding due to cancer can experience a broad spectrum of clinical manifestations, ranging from subtle neurological changes to more overt symptoms such as hydrocephalus, which necessitates immediate therapeutic intervention [7]. Hydrocephalus, in this context, often manifests due to the blockage of cerebrospinal fluid pathways by metastatic cells, leading to increased intracranial pressure and a constellation of symptoms including headaches, nausea, and changes in consciousness [8]. The approach undertaken in this study—merging VP shunt placement with reservoir installation for chemotherapy delivery—aims to streamline patient care by reducing the need for multiple surgeries. This method not only seeks to alleviate the symptoms of hydrocephalus but also to administer localized chemotherapy to treat LC directly. Given the complexity of managing patients with advanced cancer and LC, who often have limited physical reserves and a higher risk of surgical complications, the potential to minimize surgical interventions while addressing both hydrocephalus and LC could represent a significant advancement in patient care.

Patients with BM advancing to a stage necessitating ITC for LC with concurrent HC represent some of the most critically ill cancer patients. Their treatment involves a careful balance: maximizing the benefits of available therapeutic interventions while being mindful of their limited remaining physical strength as the disease progresses. Typically, ITC and the neurosurgical management of HC are considered as last-resort options. Given this context, the proposition of subjecting these already vulnerable patients to two distinct surgical interventions—one for reservoir installation for ITC and another for VPS placement for HC—raises significant concerns. Despite the procedural feasibility of each surgery, the cumulative burden can be considerable.

In scenarios where both ITC and HC treatment needs arise concurrently, or when one is present and there is a substantial risk of the other developing soon, consolidating the treatment into one surgery becomes particularly advantageous. This consolidation may not only streamline the therapeutic process but also minimize the overall stress on the patient’s system. It reflects a strategic shift towards efficiency and patient-centric care, ensuring that interventions are as non-invasive as possible while addressing both critical aspects of the patient’s condition. Therefore, this approach not only rationalizes the utilization of medical resources, but also aligns with a compassionate care model that prioritizes the patient’s quality of life and overall well-being.

The literature already mentions several other attempts to combine these therapy concepts, such as the use of additional intermediate on/off valves [8,11,21]. With the advance of programmable valves, the on–off valve has been utilized less frequently because setting a programmable valve to the maximum pressure setting before intrathecal injection is equivalent to switching off the flow and seems to be a more straightforward approach. Given the significantly reduced physical resources of the affected patients, a surgically straightforward solution seems prudent. Intraoperative prolonged assembly of multiple complex shunt components (valve, on/off switch, gravitation unit, ventricular catheter, reservoir) could lead to an increased risk of infection despite strict adherence to sterility guidelines. By omitting an additional on–off valve—as in the present study—ancillary and potentially infection-prone intraoperative VPS construction steps can be obviated. The shunt system can be effectively switched off bedside by simply increasing the flow resistance rate to a maximum in a standard shunt valve before the ITC application.

The present report describes a combination procedure consisting of a standard VPS system and a standard reservoir that arose from the imperative of these clinical considerations and has now become part of the clinical routine in our neuro-oncological specialty center. However, the vulnerable patient cohort, characterized by a poor baseline status, as indicated by a median preoperative Karnofsky performance score of 50 (IQR 40–60), could experience significant peri- and postoperative complications. No significant postoperative complications occurred beyond the expected extent [22] within the investigation of a critically and terminally ill patient cohort, so that it may cautiously be termed a safe surgical endeavor. Also, concerning further handling by the colleagues administering treatment in neuro-oncology, no significant limitations in daily application (e.g., in the context of ITC) were found. Furthermore, the modified combination technique presented herein does not require any expensive new acquisition of technical equipment—rather, it is already part of the everyday neurosurgical inventory, which certainly also contributes to the observed safety.

In the present study, the median overall survival post-surgery for the cohort was 4 months (95% CI 0–10). A comparative reference to a study by Jung et al. reveals that they reported an OS of 1.7 and 5.7 months for patients with LC without and with shunt system treatment for HC, respectively [23]. However, these purported survival benefits did not achieve statistical significance, and an OS of 2.3 months was observed in patients with LC without HC [23], indicating variable survival outcomes contingent on the presence of HC and the intervention applied. These findings highlight that the intended benefit of ventriculoperitoneal shunt implantation might not lie in extending patient lifespan but in significantly reducing the burden of symptoms caused by hydrocephalus. Notably, prevalent preoperative conditions such as lethargy, cognitive deterioration, and visual impairments, which are directly attributed to hydrocephalus, were identified. Post-surgical evaluations revealed that 86% of patients experiencing these symptoms reported noticeable improvements after undergoing shunt placement.

This enhancement in symptom management underscores the efficacy of shunt implantation in providing substantial relief, thereby elevating the overall condition for patients. The procedure demonstrates a crucial intervention that primarily serves to mitigate the discomfort and functional impairments associated with hydrocephalus, rather than offering a curative solution to the underlying malignancy. Our findings thus suggest that the strategic application of shunt implantation as a palliative measure can offer meaningful improvements in patient well-being, encompassing both observable clinical outcomes and patient-reported experiences of symptom alleviation. Therefore, this approach asserts the value of symptom-oriented interventions within the broader context of managing advanced malignancies, where enhancing the patient’s subjective condition becomes a paramount objective.

### Limitations

This study faces significant limitations. The data were collected retrospectively and depict an individual decision-making process among a very small patient group. The cohort size of consecutive patients with LC is insufficient to determine the differential effects of shunt placement alone versus the combination of intrathecal chemotherapy and shunt placement for treating malignancy-related hydrocephalus with existing LC concurrently. The diversity of underlying malignancies in the cohort further challenges outcome analysis, especially regarding potential survival benefits. Future, potentially multicentric studies may enable a differential outcome analysis focusing on survival in relation to the administration of intrathecal chemotherapy. Nonetheless, the current data offer a candid descriptive insight into the rare individual decision-making required for these terminally ill cancer patients.

## 5. Conclusions

The present study demonstrates that combining a ventriculoperitoneal shunt with an adjustable valve and a Rickham reservoir offers a viable and secure method for managing terminally ill patients affected by LC, hydrocephalus, and intrathecal chemotherapy. This integrated surgical approach effectively alleviates symptoms related to hydrocephalus and facilitates chemotherapy delivery, minimizing the overall surgical burden on patients. The observed radiological improvements and the significant reduction in preoperative symptoms, alongside a manageable rate of postoperative complications, affirm the procedure’s efficacy and safety. Furthermore, the ability of local oncology teams to independently manage shunt adjustments post-surgery underscores the practicality of this solution in a clinical setting, highlighting its potential to improve patient outcomes by simplifying treatment protocols for complex conditions.

## Figures and Tables

**Table 1:** Patient characteristics *.

Patient No	Age, Sex	Underlying Malignancy	Symptoms before VPS Surgery	Improvement of Symptoms after VPS	Used Intrathecal Chemotherapy Agent	SRC
1	51, male	gastrointestinal	H/A, lethargy, N/V, gait disturbance, urinary incontinence	none	no ITC	none
2	50, female	breast	H/A, lethargy, N/V	H/A	MTX	none
3	50, female	breast	cognitive impairment, H/A, lethargy, N/V, visual disturbance	cognitive impairment, lethargy, N/V	no ITC	none
4	63, male	lung	cognitive impairment, lethargy, visual disturbance, gait disturbance, hemiparesis, seizures	lethargy, hemiparesis	no ITC	none
5	64, male	melanoma	cognitive impairment, H/A, lethargy, gait disturbance	none	MTX	yes
6	66, female	breast	H/A, lethargy, visual disturbance, gait disturbance	H/A, lethargy, gait disturbance	MTX	yes
7	42, male	gastrointestinal	radiological HC	radiological HC	MTX	none
8	66, female	lung	H/A, lethargy, N/V	H/A, lethargy, N/V	MTX	none
9	52, male	gastrointestinal	H/A, lethargy, visual disturbance, gait disturbance	lethargy	no ITC	none
10	55, female	breast	H/A, lethargy, visual disturbance, gait disturbance	H/A, lethargy	no ITC	yes
11	60, female	breast	H/A, visual disturbance, gait disturbance	H/A	no ITC	none
12	72, female	breast	H/A, lethargy, gait disturbance	H/A, lethargy	MTX	none
13	66, female	urogenital	cognitive impairment, H/A, gait disturbance, hemiparesis	cognitive impairment, H/A, gait disturbance, hemiparesis	no ITC	none
14	65, female	breast	cognitive impairment, lethargy, gait disturbance	cognitive impairment, lethargy	no ITC	none
15	51, male	lung	cognitive impairment, gait disturbance, hemiparesis	cognitive impairment	no ITC	none
16	43, female	breast	radiological HC	radiological HC	no ITC	none

* No., number; VPS, ventriculoperitoneal shunt; SRC, shunt-related complications; H/A, headache; N/V, nausea/vomiting; ITC, intrathecal chemotherapy; HC, hydrocephalus; MTX, methotrexate.

**Table 2:** Postoperative complications.

Patient No.	Postoperative Complications	Clavien-Dindo Classification
5	data cranial wound healing disturbance, shunt infection	grade IIIb
6	data cranial wound healing disturbance without infection, abdominal catheter dislocation	grade IIIb
10	abdominal catheter dislocation, shunt infection	grade IIIb

## Data Availability

No new data were created or analyzed in this study. Data sharing is not applicable to this article.
